# Assessing Sleep Disturbance in Low Back Pain: The Validity of Portable Instruments

**DOI:** 10.1371/journal.pone.0095824

**Published:** 2014-04-24

**Authors:** Saad M. Alsaadi, James H. McAuley, Julia M. Hush, Delwyn J. Bartlett, Zoe M. McKeough, Ronald R. Grunstein, George C. Dungan, Chris G. Maher

**Affiliations:** 1 Department of physiotherapy, King Fahd Hospital of the University, The University of Dammam, Khobar, Saudi Arabia; 2 The George Institute for Global Health, Faculty of Medicine, The University of Sydney, Sydney, Australia; 3 Department of Health Professions, Faculty of Human Sciences, Australian School of Advanced Medicine, Macquarie University, Sydney, Australia; 4 The Woolcock Institute of Medical Research, The University of Sydney, Sydney, Australia; 5 Clinical and Rehabilitation Sciences, The University of Sydney, Sydney, New South Wales, Australia; 6 Department of Respiratory and Sleep Medicine, Royal Prince Alfred Hospital, Sydney, Australia; University of Regensburg, Germany

## Abstract

Although portable instruments have been used in the assessment of sleep disturbance for patients with low back pain (LBP), the accuracy of the instruments in detecting sleep/wake episodes for this population is unknown. This study investigated the criterion validity of two portable instruments (Armband and Actiwatch) for assessing sleep disturbance in patients with LBP. 50 patients with LBP performed simultaneous overnight sleep recordings in a university sleep laboratory. All 50 participants were assessed by Polysomnography (PSG) and the Armband and a subgroup of 33 participants wore an Actiwatch. Criterion validity was determined by calculating epoch-by-epoch agreement, sensitivity, specificity and prevalence and bias- adjusted kappa (PABAK) for sleep versus wake between each instrument and PSG. The relationship between PSG and the two instruments was assessed using intraclass correlation coefficients (ICC 2, 1). The study participants showed symptoms of sub-threshold insomnia (mean ISI = 13.2, 95% CI = 6.36) and poor sleep quality (mean PSQI = 9.20, 95% CI = 4.27). Observed agreement with PSG was 85% and 88% for the Armband and Actiwatch. Sensitivity was 0.90 for both instruments and specificity was 0.54 and 0.67 and PABAK of 0.69 and 0.77 for the Armband and Actiwatch respectively. The ICC (95%CI) was 0.76 (0.61 to 0.86) and 0.80 (0.46 to 0.92) for total sleep time, 0.52 (0.29 to 0.70) and 0.55 (0.14 to 0.77) for sleep efficiency, 0.64 (0.45 to 0.78) and 0.52 (0.23 to 0.73) for wake after sleep onset and 0.13 (−0.15 to 0.39) and 0.33 (−0.05 to 0.63) for sleep onset latency, for the Armband and Actiwatch, respectively. The findings showed that both instruments have varied criterion validity across the sleep parameters from excellent validity for measures of total sleep time, good validity for measures of sleep efficiency and wake after onset to poor validity for sleep onset latency.

## Introduction

Low back pain (LBP) is a common health condition; it has a lifetime prevalence of 40%, and a point prevalence (at any point in time) of 20% [1]. LBP is associated with both physical and psychological consequences, for example disability, depression and anxiety [2]. Studies have also found that between 50–60% of patients with LBP report sleep disturbance [3], [4]. A recent systematic review found that people with chronic LBP report increased duration to sleep onset, reduced total sleep time, and lower sleep efficiency [5].

Sleep disturbance in patients with LBP is associated with psychological distress, physical disability [6], fatigue and day-time sleepiness [7]. Patients with LBP who complain of sleep disturbance have been found to experience more severe pain [4] and are more likely to be hospitalized for their LBP than those without sleep problems [8]. The literature suggests that there is a bidirectional relationship between disturbed sleep and intensity of pain. (i.e. pain may lead to the reporting sleep disturbance and poor sleep may cause or exacerbate the pain) [9]. It is plausible therefore that disturbed sleep is likely to adversely impact LBP management. Moreover, the consequences of sleep disturbance can hinder treatment effectiveness. For example, exercise therapy, used to reduce pain, improve function and enhance return to work [10], is a critical component of LBP management. Clinicians prescribe exercise therapy for approximately half of their patients with LBP [11]. The effects of sleep disturbance such as fatigue are likely to hinder exercise performance and consequently lead to poorer treatment outcomes. For these reasons the assessment of sleep disturbance in patients with LBP is an important clinical and research question.

The gold standard for assessing sleep quality is Polysomnography (PSG). However, due to its complexity and expense it is infrequently used in research on sleep quality in patients with LBP. Most studies reporting on sleep quality and LBP have used data from self-report questionnaires [5]. Self-report measures however correlate poorly with PSG and therefore estimates of sleep parameters in patients with LBP may not be accurate [12]. Newly developed portable instruments provide a less expensive, objective alternative to PSG and are a potentially more accurate method of measuring sleep quality in a free-living environment than self-report measures [13]. A commonly used portable method is actigraphy, in which a small device containing an accelerometer to detect limb movement is worn on the wrist or ankle. A mathematical algorithm is used to estimate sleep/wake episodes. The accuracy of actigraphy to evaluate sleep parameters has been investigated by comparison with PSG in several medical conditions and for some sleep disorders, but not for the LBP population [14]–[16].

In addition to accelerometry, the BodyMedia SenseWear Armband, acquires other physiological signals including skin temperature, galvanic skin response and heat flux which are thought to be important in determining sleep/wake cycles [17]. This instrument may therefore provide a more accurate assessment of sleep quality than actigraphy alone [18]. Further, because the Armband is worn on the upper arm interference from fine limb movement associated with limb dominance is minimised. Despite the potential superiority of the Armband, only 4 studies have investigated its accuracy in detecting sleep/wake episodes measured by polysomnography. These studies were performed in healthy children and adolescents [19], healthy volunteers placed on hypnotics or placebo [20] and sleep apnea [21], [22]. There was variability in findings across the studies with the Armand able to detect patients' sleep with more accuracy than detecting wake episodes. Only one study examined a different actigraphy device in parallel with the Armband [20].

Although a number of studies [6], [23]–[25] have employed actigraphy for the assessment of sleep disturbance in patients with LBP, the accuracy of the instrument for this population is uncertain. In particular, there is some evidence that patients with LBP exhibit higher body activity during sleep than those without LBP [26], which might adversely affect the accuracy of the actigraphy in detection of an individual's sleep/wake episodes. This casts some doubt on whether findings of previous validity studies in other conditions generalise for the LBP population. Therefore, the aim of the current study was to determine the criterion validity of the actigraph and the BodyMedia SenseWear Armband for measuring sleep parameters in a sample of patients with LBP by comparing the instruments' recordings of sleep/wake to those of the PSG. A secondary aim was to investigate whether the additional physiological measures provided by the Armband increase the accuracy of sleep parameters compared with accelerometry alone (i.e. actigraph).

## Materials and Methods

This was a cross-sectional study conducted between March 2010 and June 2011. The study protocol was approved by the University of Sydney Human Research Ethics Committee, Australia (09-2009/12100). All participants signed informed consent forms before participation in the study. Participants were compensated for their time and transportation expenses.

### Participants

Participants with non-specific LBP were recruited from physiotherapy clinics in the Sydney metropolitan area and from the community through advertising. The inclusion criteria were: patients aged between 18 and 79 years with a primary complaint of LBP (pain between the 12^th^ rib and buttock crease) with or without leg pain (pain radiating to the lower limb “sciatica”) and possessing sufficient fluency in the English language to understand and respond to instructions. Exclusion criteria were: LBP caused by a serious spinal pathology, according to medical/physiotherapy evaluation or patient report; nerve root compromise (evidenced by at least two of myotomal weakness, dermatomal sensory loss, or hypo-reflexia of the lower limb reflexes); spinal surgery within the preceding 6 months; previously diagnosed with a sleep disorder for which they were receiving care; and patients receiving care for a mental health condition.

### Procedures

Participating physiotherapists informed patients about the study. If a patient indicated an interest in participating, the physiotherapist provided them with comprehensive information about the study procedures and then passed on their contact details to the study researcher. The researcher contacted the patient, screened the patient for eligibility, and arranged a time to meet eligible patients at the sleep laboratory. Potential participants from the community were provided with comprehensive information about the study through the post or electronic mail. Those who showed an interest in participating were then contacted and screened for inclusion by the study researcher. All who met the eligibility criteria met the study researcher at the sleep laboratory.

The sleep assessments were conducted at the sleep laboratory of the Woolcock Institute of Medical Research, the University of Sydney, Australia. The study researcher, who was a trained physiotherapist, met the participants to screen for neurological signs, obtain informed consent, collect basic demographic information (including age; gender; body mass index; nationality; level of education; whether currently seeking care) and participant's clinical condition (including pain intensity; pain duration; disability; psychological distress; fatigue –see Table S1). Although participant's clinical profiles were not included in the analysis, they were assessed to describe the study sample. At the end of the interview the researcher fitted the Armband and the Actiwatch and provided instructions on how to avoid getting the Armband and Actiwatch wet during bathing/showering. A sleep laboratory technician then carried out the overnight PSG study.

### Sleep measurement

Each participant had measures of sleep parameters taken with the Armband and Actiwatch and also with PSG, which is considered the criterion measure, while sleeping overnight in the sleep laboratory. The assessed sleep parameters were: total sleep time (TST); sleep onset latency (SOL); sleep efficiency (SE) and wake after sleep onset (WASO). These parameters were calculated as below:

TST: the total number of minutes scored as sleep from lights out to lights on.

SOL: the total number of minutes scored as awake beyond lights out prior to sleep onset.

SE: the ratio of minutes spent asleep to total minutes in bed, X 100 (expressed as percentage).

WASO: the total minutes scored as wake after sleep onset before lights on.

The evaluation of these sleep parameters (TST, SOL, SE and WASO) was based on “lights off” and “lights on” time according to the PSG recording. Lights off was the time that the patient started trying to sleep (start of PSG) and lights on was the time that the patient was awakened (end of PSG), as recorded by the sleep laboratory technician.

### Armband

An Armband (SenseWear-Pro3, BodyMedia Monitoring System, Pittsburgh PA, USA) was attached on the right upper arm during testing according to the manufacturer's instructions. The software (SenseWear Professional Software version 6.1) used average variations in body movements, differential and proportional changes in heat-flux and skin temperature and the galvanic skin response to score each 60 second time epoch as either sleep or awake [18].

### Actiwatch

Following the manufacturer's instructions the Actiwatch (Actiwatch 2; Philips Respironics, Murrysville PA, USA) was attached to the non-dominant wrist during testing. Epoch length was set at 30 secs to match the PSG setting. The data were downloaded and analysed using ActiWare (R) software version 5.52.0003 (Philips Respironics, Murrysville, PA, USA). The standard factory-default algorithm was used for sleep interval detection. The parameters were: wake threshold set as “medium” and the sleep interval detection algorithm set as “immobile minutes.” Immobile minutes for sleep onset and end of sleep were set at 10 minutes.

The software scored each epoch as either asleep or awake by evaluating the level of activity compared to the immediate prior and subsequent epochs of activity (±2 min). The threshold value was set to medium (wake threshold value = 40). The medium threshold value has been validated in a previous technical report [27]. If the number of activity movements (count) exceeded the threshold, the epoch was scored as wake. If activity counts fell below or were equal to the threshold, the epoch was scored as sleep [28].

### Polysomnography (PSG)

Overnight attendance in-laboratory polysomnography (PSG) was performed in a university-based research sleep laboratory. The contemporary standard technique was used for the recording and included measurements obtained from the following: electroencephalogram (EEG) central channels (C3-M2, C4-M1) and occipital channels (O1-M2, O2-M1), bilateral electrooculogram (EOG), chin electromyogram (EMG), bilateral tibialis anterior EMG, Lead II electrocardiogram (ECG), nasal air-flow (pressure derived), fingertip pulse oxygen saturation (SpO2), snoring using a PTAF lite pressure transducer sensor, and body position using a body position sensor. The PSG study was performed using the Sandman system (Tyco Healthcare, Colorado, USA). Sleep staging was scored using current American Academy of Sleep Medicine criteria AASM [29]. Each epoch was assigned a stage of sleep or wake on the basis of the EEG, EOG and EMG channels. Respiratory events and arousals were scored according to standard AASM (alternative hypopnoea definition) and the American Sleep Disorders Association criteria ASDA [30], respectively. The Apnea-Hypopnea index (AHI) was calculated by dividing the total number of apneas and hypopneas by the total sleep time (hours).

As synchronisation of time is critical to accurately compare the device and PSG epochs optimal matching between the “lights off” and “lights on” time agreement was tested a number of times within a ±2 min time range. The peak agreement value was used in the final analysis [31].

### Data Analysis and Statistical Methods

#### Epoch by epoch sleep/wake agreement

We evaluated the criterion validity of the Armband and Actiwatch by comparing sleep/wake episodes reported by these devices to the sleep/wake episodes reported by PSG. For each participant we calculated epoch by epoch sleep/wake agreement using the Prevalence and Bias-Adjusted Kappa (PABAK) [32], sensitivity and specificity. Agreement, sensitivity and specificity calculations were based on results of 2×2 table, where PSG is considered the reference standard and the two portable sleep instruments are considered as index tests.

The PSG and the Actiwatch evaluate individual's sleep in 30 seconds intervals, whereas the Armband evaluates sleep in 60 seconds intervals, called sleep epochs. The Armband's epoch length is calibrated by the manufacturer and could not be altered. Therefore, to compare the 60-second epochs of the Armband to the 30-second epochs of the PSG each Armband's epoch score was harmonised to 60 seconds before the analysis.

#### Evaluation of sleep parameters

Sleep parameters (total sleep time TST, sleep onset latency SOL, sleep efficiency SE, wake after sleep onset WASO) estimated by the Armband and Actiwatch described above, were compared to sleep parameters estimated by the PSG (the reference standard measure) in a parallel form of reliability using the intraclass correlation coefficients (ICC 2, 1) with two-way model using single measure and absolute agreement, (reported with 95% confidence intervals (CIs). In addition, scatter plots, regression analyses and Bland and Altman plots were used for comparison purposes. We chose this approach because it is generally agreed that there is no single statistical procedure that adequately covers this issue [33], [34]. To describe the criterion validity of the continuous sleep parameters we compared the obtained ICC values to the benchmarks proposed by Fleiss for excellent reliability (>0.75), fair to good reliability (0.4 to 0.75) and poor reliability (<0.4) [35]. Finally, to compare the criterion validity of the Armband to that of the Actiwatch we compared the 95% CI of the obtained statistics.

Statistical analyses were conducted using SPSS version 17 (SPSS Inc., Chicago, IL) and MedCalc for Windows, version 9.5.0.0 (MedCalc Software, Mariakerke, Belgium).

## Results

### Characteristics of study participants

Fifty patients with non-specific LBP participated in the study. The sample's demographic information and clinical description are shown in Table 1. The majority (92%) of the sample had chronic LBP with a mean (SD) pain intensity of 4.12 (1.9) on a 0–10 scale. Twenty-eight participants (56%) were seeking care for their LBP. The sample's mean (SD) weight was 76.7 (20.9) kg, with a body mass index of 25.7 (5.2) kg/m^2^.

**Table 1:** Characteristics of study participants.

	Mean (SD)
Age (years)	42.7 (15.15)
BMI (kg/m^2^)‡	25.7 (5.21)
Pain intensity NRS (0–10)*	4.2 (1.90)
Low back symptoms duration (year)	10.6 (9.92)
Disability (RMDQ) (0–24)#	8.48 (5.49)
Depression (DASS-21) (0–21)$	10.1 (10.1)
Anxiety (DASS-21) (0–21)	8.8 (7.90)
Stress (DASS-21) (0–21)	14.8 (9.74)
Fatigue (FSS) (0–63)†	35.3 (12.63)
Sleep quality (PSQI) (0–21)**	9.2 (4.27)
Insomnia severity (ISI) (0–28)≠	13.2 (6.36)
Day-time sleepiness (ESS) (0–24)α	8.2 (5.55)

	N (%)
Female gender	24 (48)
Seeking care	28 (56)
University degree holder	25 (50)

‡ BMI, body mass index;

*NRS, numerical rating scale (pain right now);

# RMDQ-24, Roland and Morris disability questionnaire: 24-item version;

$ DASS-21, depression, anxiety and stress scale: 21-item version;

† FSS, fatigue severity scale.

**PSQI, Pittsburgh sleep quality index;

≠ ISI, insomnia severity index;

α ESS, Epworth sleepiness scale.

The psychological distress assessment, using the DASS-21, indicated that the majority of participants were within normal level of depression, anxiety and stress. Thirty two (64%) participants scored within normal levels for depression (total DAAS-21 depression subscale <9), 28 (56%) participants scored within normal levels for anxiety (total DAAS-21 anxiety subscale <7) and 27 (54%) participants scored within normal levels for stress (total DAAS-21 stress subscale <14). Likewise, the fatigue assessment showed that 27 (54%) participants had fatigue scores within the normal levels (i.e. total FSS <36). After completing the PSG testing a sleep physician diagnosed 4 (8%) participants with severe obstructive sleep apnea (OSA), 3 (6%) participants with moderate OSA, and 18 (36%) participants with mild OSA. Self-reported sleep measurement showed that 37 (74%) participants had poor sleep quality according to the Pittsburgh Sleep Quality Index PSQI (i.e. >5) [36], [37], and 32 (64%) participants had symptoms of clinical insomnia according to the Insomnia Severity Index ISI (i.e. >14), [38], [39]. Similarly, 38 (76%) participants showed evidence of excessive day-time sleepiness as measured by the ESS (i.e. <10). Two patients used escitalopram, an oral drug used for treating depression and/or generalized anxiety disorder.

For the duration of the PSG recording, all 50 participants wore the Armband, while only 33 participants wore the Actiwatch, due to limited Actiwatch availability. The mean (SD) time spent in bed during the PSG recording was 7.13 (1.2) hrs, with mean (SD) total sleep time of 6.02 (1.0) hrs, mean (SD) sleep onset latency 15.19 (14.2) mins, mean (SD) wake after sleep onset of 47.30 (35.7) mins and overall sleep efficiency of 84.5%.

### Epoch by epoch sleep/wake agreement

Table 2 shows the sensitivity, specificity, PABAK, and agreement (proportion and 95% CI) results derived from epoch by epoch comparison between the Armband, Actiwatch and PSG.

**Table 2:** Epoch-by-Epoch Sleep/Wake Agreement between Armband, Actiwatch and PSG.

Measure	Armband n = 50	Actiwatch n = 33
	Mean (95% CI)	Mean (95% CI)
Sensitivity	0.90 (0.88 to 0.93)	0.90 (0.88 to 0.93)
Specificity	0.54 (0.46 to 0.62)	0.67 (0.60 to 0.74)
Agreement	0.85 (0.81 to 0.88)	0.88 (0.86 to 0.90)
PABAK	0.69 (0.63 to 0.75)	0.77 (0.73 to 0.81)

Sensitivity is the proportion of “sleep” epochs as defined by PSG that were judged as “sleep” by Armband/Actiwatch. Specificity is the proportion of the “wake” epochs as defined by PSG that were judged as “wake” by Armband/Actiwatch. Agreement is proportion of epochs where there was agreement between PSG and instrument. PABAK is the prevalence and bias adjusted kappa.

The Armband and Actiwatch both had sensitivity (i.e. detecting sleep) of 0.90. Specificity (i.e. detecting being awake) for the Armband and the Actiwatch was 0.54 and 0.67 respectively. Both instruments demonstrated a high level of observed agreement (i.e. detecting both sleep and being awake), 85% for the Armband and 88% for the Actiwatch. The prevalence-and bias-adjusted kappa (PABAK) measurement showed that both instruments have high level of agreement with the PSG in detection of sleep and wake, 0.69 and 0.77 for the Armband and for the Actiwatch, respectively.

### Continuous measures of sleep

**Figure 1:**
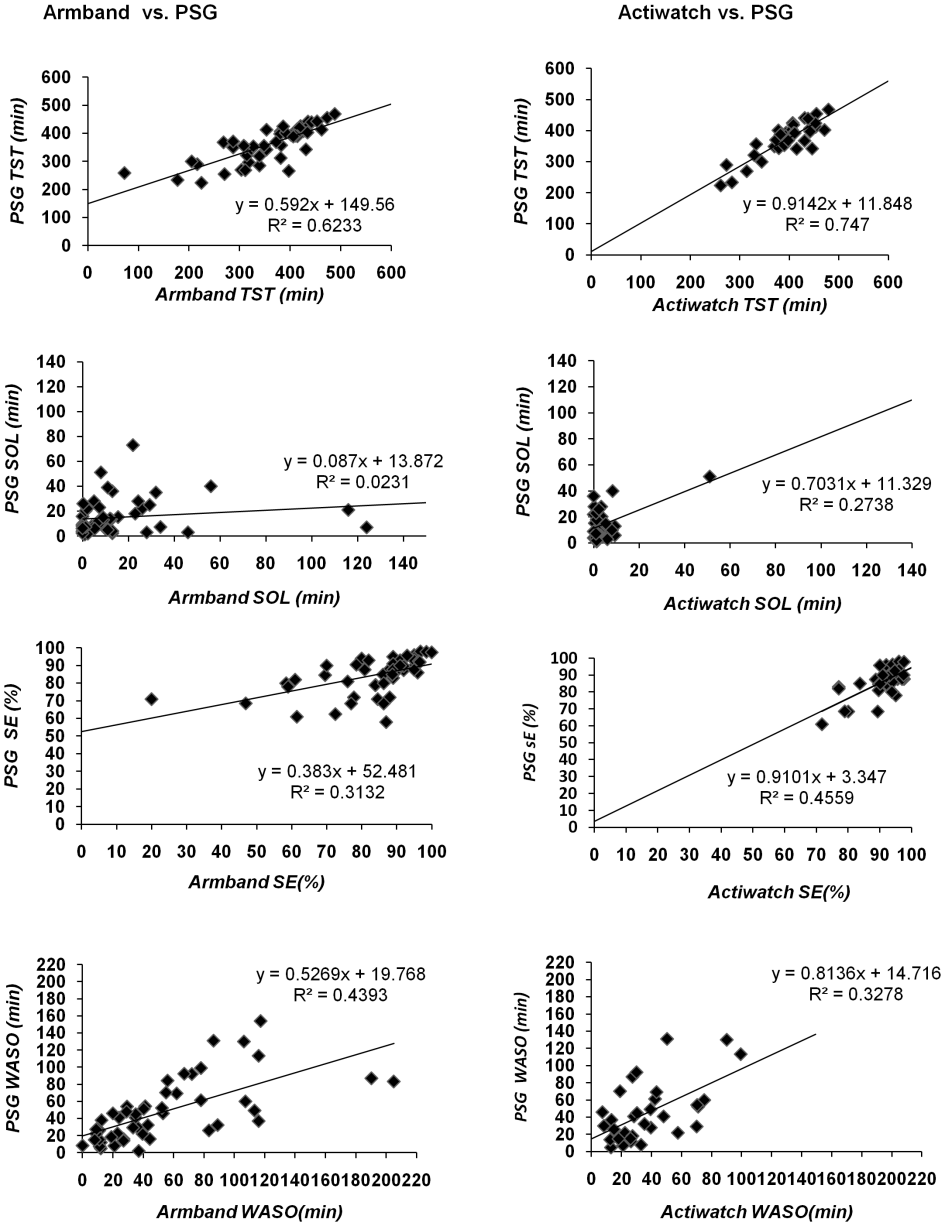
Scatter plots for sleep parameters association evaluated by Armband and Actiwatch compared to PSG. The horizontal axis represents instrument's estimation of sleep parameters: TST, total sleep time; SOL, sleep onset latency; SE, sleep efficiency and WASO, wake after sleep onset. The vertical axis represents these sleep parameters estimated by the reference standard, the PSG. Each square represents data from each participant.

Descriptive statistics for the continuous measures of sleep parameters derived from the PSG, Armband and Actiwatch, and the ICC values are presented in Table 3. The criterion validity, as reflected in the ICC values, are similar for the Armband and Actiwatch but varied across the sleep parameters from excellent validity for measures of total sleep time, good validity for measures of sleep efficiency and wake after onset to poor validity for sleep onset latency. With exception of a few extreme scores, the scatter plots and regression analyses in Figure 1 are consistent with the ICC analyses; again showing that the two instruments have a similar relationship with PSG, but the strength of the relationship varied substantially across the four continuous sleep measures.

**Table 3:** Sleep parameters' comparison between the Armband, Actiwatch and PSG.

Distribution of scores: Mean (SD) & [Range]				Parallel forms' reliability ICC (2,1) with (95% CI)	
Sleep parameter	PSG	Armband	Actiwatch	Armband vs. PSG	Actiwatch vs. PSG
TST (mins)	361.60 (62.21)	358.37 (83.04)	395.31 (56.65)	0.76 (0.61 to 0.86)	0.80 (0.46 to 0.92)
	[223.00–468.30]	[72.00–488.00]	[261.30–479.30]		
SOL (mins)	15.19 (14.23)	15.23 (24.98)	4.46 (8.80)	0.13 (−0.15 to 0.39)	0.33 (−0.05 to 0.63)
	[2.00–73.00]	[0.00–124.00]	[0.00–51]		
SE (%)	84.49 (10.28)	83.59 (15.02)	90.54 (6.67)	0.52 (0.29 to 0.70)	0.55 (0.14 to 0.77)
	[58.00–98.00]	[20.00–100.00]	[71.70–97.60]		
WASO (mins)	47.30 (35.76)	52.26 (44.99)	37.06 (24.05)	0.64 (0.45 to 0.78)	0.52 (0.23 to 0.73)
	[2.00–154.00]	[0.00–205.00]	[7.30–99.30]		

ICC, intraclass correlation coefficient: two-way model using single measure and absolute agreement; CI: confidence interval; mins, minutes; TST, total sleep time; SOL, sleep onset latency; SE, sleep efficiency; WASO, wake after sleep onset.

### Comparative performance of the two measures

There was no evidence that the Armband had greater criterion validity than the Actiwatch with the 95% CIs for the PABAK and ICC statistics overlapping.

Results from the Bland and Altman plots can be found in the supporting information section (Figure S1). These plots show the agreement between the instruments and the PSG for each sleep parameter by assessing the difference between a PSG sleep parameter and the instrument sleep parameter against the PSG sleep parameter. These plots show similar performance for the two measures.

## Discussion

Both the Armband and actigraph provided valid measures of total sleep time, sleep efficiency and wake after sleep onset but not sleep onset latency. As the Armband does not appear to have superior criterion validity to the actigraph we conclude that the parameters sampled by the Armband do not provide additional accuracy in identifying sleep/wake epochs in the way that they are measured by this device.

To our knowledge, this is the first study to evaluate the validity of actigraphy in detecting sleep/wake in patients with LBP. The study findings are consistent with results from recent systematic reviews that investigated the role of actigraphy in sleep/wake detection for other health conditions [15], [16]. These reviews have reported that actigraphy is sensitive in detecting sleep episodes, however, wakefulness detection remains somewhat problematic. Additionally, we have confirmed that the estimation of sleep onset latency is a limitation of actigraphy. This limitation may be attributed to the nature of the accelerometry which is based on body mobility detection rather than body physiological changes, as in the case of PSG [40]. Nevertheless, investigation of different types of sleep algorithms with different activity sensitivity may overcome these limitations. For example, a recent study found that lowering the actigraphy threshold to 5 minutes of immobility, rather than the standard 10 minutes, improved the detection of sleep onset latency [41].

Our study findings suggest that the Armband and the Actiwatch are useful objective tools to assess sleep parameters in patients with LBP (total sleep time, sleep efficiency, and wake after sleep onset). As these instruments are portable we conclude that they are likely to be useful for assessing sleep in a naturalistic setting. If the accurate assessment of sleep onset latency is of primary importance, researchers or clinicians could consider other devices such as the “Sleep Switch” [42], which has been found to be very strongly associated with sleep onset latency of PSG [43].

This study had several strengths. It is the first evaluation of the validity of the Armband to assess sleep parameters and the first to evaluate actigraphy in a group of patients with LBP by comparing the instruments to the widely accepted gold standard of sleep/wake detection, PSG. The study sample was recruited from both the community and primary care clinics and therefore forms a sample representative of those seeking care as well as those not currently seeking care for their LBP. Finally, we followed current recommended methods for conducting and analysing portable instruments validation against PSG [15], [34].

This study also had some limitations. First, our data were collected in a sleep laboratory setting, and therefore, may not generalize to a home environment. Further research to validate these instruments using a home portable PSG are potentially worthwhile. Second, PSG data and Actiwatch data were collected in 30-sec epochs and the Armband data were collected in 60-sec epochs. This difference may have reduced the potential for agreement for the Armband with the PSG. Third, inspection of the plots (Figure 1) identified several outliers in the data. As these cases may exert undue influence on the results, we conducted a post-hoc sensitivity analysis by removing them and re-running the statistical analyses. However, since this did not change the results, we retained these cases in the analysis. In addition, the analysis showed the 95% confidence intervals are often wide and should also be taken into consideration when interpreting the study findings. For example, the point estimate for the ICC for total sleep time by Actiwatch is 0.80, which is relatively strong (Table 3). However, the lower bound of the 95% CI is 0.46 which is only moderate and reflects some uncertainty with the results. Finally, there are a variety of sleep/wake algorithm modes with different activity sensitivities (i.e. low, medium and high). In this study we used the medium threshold setting, the commonly used threshold [44]. However, we acknowledge that other settings might improve the accuracy of the device. Thus, future research is needed to identify the optimal threshold setting to detect sleep/wake in this group of patients. Further investigation is also needed to assess the accuracy of actigraphy in detecting change (responsiveness) in sleep quality in patients with LBP that might occur following intervention.

## Supporting Information

Figure S1 **Bland and Altman Plots of Difference vs. PSG Score for Sleep Parameters Evaluated by Armband/Actiwatch against PSG.** TST, total sleep time; SOL, sleep onset latency; SE, sleep efficiency; WASO, wake after sleep onset.
(TIFF)

Table S1 **Construct and description of clinical assessment measures.**
(DOCX)
