# Supplementary material for: Assessing Sleep Disturbance in Low Back Pain: The Validity of Portable Instruments
Source: PLoS One. 2014 Apr 24;9(4):e95824. doi: 10.1371/journal.pone.0095824 (PMC3998977; doi:10.1371/journal.pone.0095824)
Supplement: Table S1 — Construct and description of clinical assessment measures. (DOCX) [file pone.0095824.s002.docx]

| **Table S1. Construct and description of clinical assessment measures** | | |
| --- | --- | --- |
| Measure | Construct | Description |
| RMDQ^a^ | Disability | The RMDQ is 24-item self-administered questionnaire designed to measure the effect of low back pain on patient’s normal activities of daily living. The participant indicates their agreement or disagreement with each statement (0= do not agree) or (1= agree). The RMDQ total score is obtained by summing the total items giving a range from 0 to 24, where the higher score indicates more severe disability. |
| DASS-21^b^ | Depression, anxiety and stress | The DASS is 21-item self-report questionnaire designed to measure the severity of depression, anxiety and stress over the past week. The participant reports his/her experience of emotional distress by selecting to what extent each item applies him/her (0= did not apply to me at all; 3= applied to me very much, or most of the time). The DASS-21 total score is obtained by summing items of each scale (depression, anxiety and stress). |
| FSS^c^ | Fatigue | The FSS is 9-item self-report questionnaire designed to measure the impact of fatigue, with 7-point likert scale. The participant rates his/ her severity of fatigue symptoms by selecting one of the 7 points on a 7 point likert scale (1= disagree; 7= agree). The total score is obtained by summiting the item scores. A total score of less than 36 suggests no fatigue, while a total score of 36 or more suggests fatigue and further evaluation by a physician is needed. |
| ^a^Roland and Morris Disability Questionnaire. *Reference*: Roland M and Morris R. (1983) A study of the natural history of back pain. Part I: development of a reliable and sensitive measure of disability in low-back pain. Spine;8:141-4. ^b^Depression, Anxiety and Stress Scale. *Reference*: Brown TA, Chorpita BF, Korotitsch W, Barlow DH. (1997) Psychometric properties of the Depression Anxiety Stress Scales (DASS) in clinical samples. Behav Res Ther 35:79-89. ^c^Fatigue Severity Scale. *Reference*: Krupp LB, LaRocca NG, Muir-Nash J, Steinberg AD. (1989) The fatigue severity scale. Application to patients with multiple sclerosis and systemic lupus erythematosus. Arch Neurol 46:1121-3. | | |
